# Critical care transport in the time of COVID-19

**DOI:** 10.1017/cem.2020.400

**Published:** 2020-05-13

**Authors:** Homer Tien, Bruce Sawadsky, Michael Lewell, Michael Peddle, Wade Durham

**Affiliations:** * Ornge, Mississauga, ON; † Department of Surgery, Sunnybrook Health Sciences Centre, University of Toronto, Toronto, ON

**Keywords:** COVID-19, EMS, infectious diseases, microbiology, prehospital

## Abstract

Critical care transport organizations are nimble, operationally focused institutions that can aid in managing crises. Ornge provides air ambulance and critical care transport services to Ontario. From 12 bases, Ornge operates four PC-12 Next Generation fixed wing (FW) aircraft, eight AW-139 rotary wing (RW) aircraft, and four critical care land ambulances (CCLA) on a 24/7 basis. Ornge also contracts with private air carriers to provide lower acuity air ambulance services. Ornge performs over 20,000 patient-related transports annually. We discuss Ornge's approach to preparing for the coronavirus disease 2019 (COVID-19) pandemic, and identify potential unconventional roles.

Critical care transport organizations are nimble, operationally focused institutions that can aid in managing crises.^1^ Ornge provides air ambulance and critical care transport services to Ontario. From 12 bases, Ornge operates four PC-12 Next Generation fixed wing (FW) aircraft, eight AW-139 rotary wing (RW) aircraft, and four critical care land ambulances (CCLA) on a 24/7 basis. Ornge also contracts with private air carriers to provide lower acuity air ambulance services. Ornge performs over 20,000 patient-related transports annually. We discuss Ornge's approach to preparing for the coronavirus disease 2019 (COVID-19) pandemic, and identify potential unconventional roles.

## ORNGE DURING COVID-19

As of April 30, 2020, Ornge has organized transport for and/or transported 325 patients with either a confirmed case of or under investigation for COVID-19. A total of 52.3% of these were completed by CCLA, 28.9% were completed by FW aircraft, and 16.6% were completed by RW aircraft. Of these, 71% required oxygen therapy, approximately 1% received oxygen by high flow nasal cannula (HFNC), 59% were intubated and being mechanically ventilated, approximately 1% were transported prone, and approximately 1% were transported on extracorporeal membrane oxygenation. The average duration of these transports was 115 minutes. During this time, no staff members tested positive for COVID-19.

On March 11, 2020, the World Health Organization declared a global pandemic.^2^ On February 28, Ornge activated its pandemic committee. The pandemic committee's priorities include ensuring staff safety, developing surge capacity, and responding to urgent, unconventional requests for assistance.

## MAINTAINING OPERATIONAL READINESS BY ENSURING STAFF SAFETY

Northern communities are relatively isolated from COVID-19. To mitigate the risk of spread to northern communities and northern Ornge bases from the south, we instituted a strict travel ban. We instituted a work from home program for non–front-line staff, and split our Operational Communications Centre (OCC) staff into two separate groups that worked out of two different locations.

### 1. Protecting staff physical health

#### A. COVID-19 screening and personal protective pquipment

Protecting our staff has been our top priority. Early in the pandemic, Ornge implemented screening using the Provincial Transfer Authorization Centre (PTAC). PTAC offers an online tool to screen for COVID-19. All transport providers are warned of potential COVID-19 transfers before transport. Ornge decided early during the pandemic to use airborne precautions for all potential COVID-19 cases. Ornge paramedics are unable to change from droplet to airborne precautions while in an aircraft or vehicle, before performing an aerosol-generating procedure (AGP).

Ornge also procured reusable N95 facemasks (3M 6500) with disposable filters as well as washable water-resistant gowns. We have not adopted the use of powered air-purifying respirators (PAPRs), because of the increased risk of contamination during doffing,^3^ and although PAPRs have a higher protective factor compared with N95 respirators, there is no definitive evidence that PAPRs reduce the likelihood of viral transmission in the setting of potential airborne spread.^4^ Furthermore, significant training burden is required to maintain competency, and there remains difficulty with implementation in the aviation environment.

#### B. Aircraft/vehicle suitability

The Centers for Disease Control and Prevention (CDC) provides guidance on airframe selection for transporting severe acute respiratory syndrome (SARS) and Middle East respiratory syndrome (MERS) coronavirus (CoV) patients.^5,6^ Among other recommendations, the CDC recommends aircraft with front-to-aft cabin air flow and a cabin separate from the cockpit for transporting these infectious patients. In aircraft with uncontrolled interior air flow, the CDC suggests that all personnel wear N-95 masks. Additionally, the CDC offers recommendations for ventilation requirements in hospitals for patient care areas.^7^ For airborne infection isolation rooms, the CDC recommends a minimum of 6–12 total air changes per hour (ACH).^7^

In our FW aircraft, the cockpit is not separate from the cabin, the air flow is aft-to-front, and the interior cabin air exchange is approximately 10 ACH. For our RW aircraft, cabin air flow varies from 9 to 36 ACH. All but two of our RW aircraft have a fixed wall installed, resulting in independent cockpit and cabin ventilation. Our CCLA assets are capable of 24 ACH per hour and the front driver's compartment is separate from the patient compartment. As such, we preferentially transport COVID patients using our CCLA assets. Our CCLA assets are capable of more than adequate ACH, fewer personnel are exposed to the risk of infection, and the front driver's compartment is separate from the patient compartment.

We have also restricted the use oxygen by high flow nasal cannula (HFNC) to only CCLA assets and temporarily halted the use of noninvasive ventilation (NIV). There is emerging evidence to support the use of HFNC and NIV to prevent mechanical ventilation in COVID-19 patients.^8,9^ However, there is substantial fear among health care workers about the increased risk of infection transmission from using HFNC and NIV,^10^ although unsubstantiated with evidence. In cases where air transport is required because of geography, Ornge will require these patients to be mechanically ventilated. Ornge will be participating in engineering research trials looking at the risk of droplet and aerosol dispersion using HFNC and NIV in our airframes based on their current airflow dynamics.

#### C. Ambulance Decontamination

Given that SARS-CoV, the virus that causes COVID-19, can live for as long as 72 hours on surfaces,^11^ decontamination of ambulances is particularly important. Decontamination represents a high-risk activity requiring PPE for health care workers.^12^ For the COVID-19 pandemic, we have now adopted the use of a vaporized hydrogen peroxide disinfection system (Nocospray, Montreal), which minimizes health care worker exposure.

### 2. Protecting staff mental health

Communication has been the touchstone of all our efforts to mitigate the anxiety and mental health consequences of working during this pandemic. We have held weekly town-halls, sent out twice-weekly communications, have a dedicated COVID-19 information page on our internal website, and have included staff family members in our communications. We have activated our peer support cadre to run “Village Halls” to address base-level issues. We continue to address specific occupation-specific concerns about working in the COVID-19 environment (i.e., pilot v. paramedic). Finally, after transporting COVID-19 patients, Ornge crews can take an operational pause to decompress and debrief. As of April 30, 2020, a total of 89 operational pauses have been instituted. The average duration of an operational pause has been 60 minutes.

## SURGE CAPACITY

**Figure 1.**
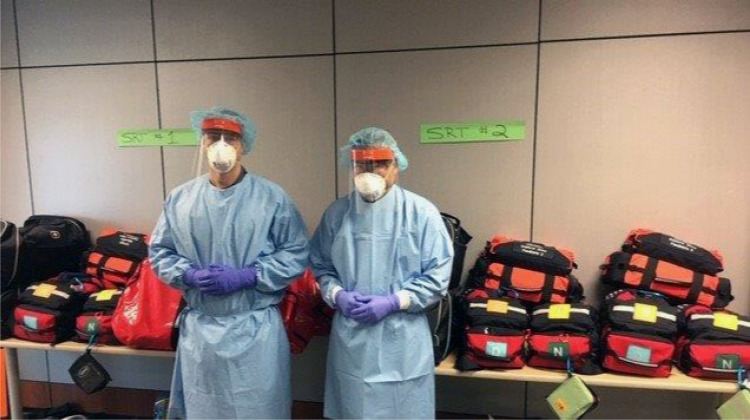
Ornge surge response team with their kits.

The Ontario Patient Care and Transportation Standards requires each ambulance be staffed with two paramedics.^13^ Under the prepandemic staffing matrix, Ornge required that a critical care paramedic be paired with a second critical care (or advanced care flight) paramedic to provide critical care transport. For the pandemic, Ornge changed the staffing matrix so that critical care transport can be performed by one critical care paramedic and one primary care paramedic, thereby doubling capacity. As well, Ornge has solicited paramedic volunteers to form a COVID-19 Ornge Surge Response Team (OSRT). Forty-six Ornge paramedics from across the province volunteered. They could be dropped off at any facility to help with airway management and mechanical ventilation pending transport. The team's deployment kit has the equipment and medications to be able to function independently, and includes a fully equipped airway management bag, a portable mechanical ventilator, monitors, medications, and infusion pumps (Figure 1). Their equipment is also compatible with the transport equipment of all municipal land ambulances.

### 3. Northern community surge plan

For northern communities, Ornge has augmented three remote health care facilities with additional ventilators from our own stockpile. As well, Ornge can increase our normal FW response with OSRT paramedics in all other available FW aviation assets. Our private air carriers traditionally only transport one patient at a time. However, we have modified our operating practice to allow for two ventilated patients to be transported in select private air carrier aircraft with our OSRT paramedics.

### 4. Southern critical care transport

**Figure 2.**
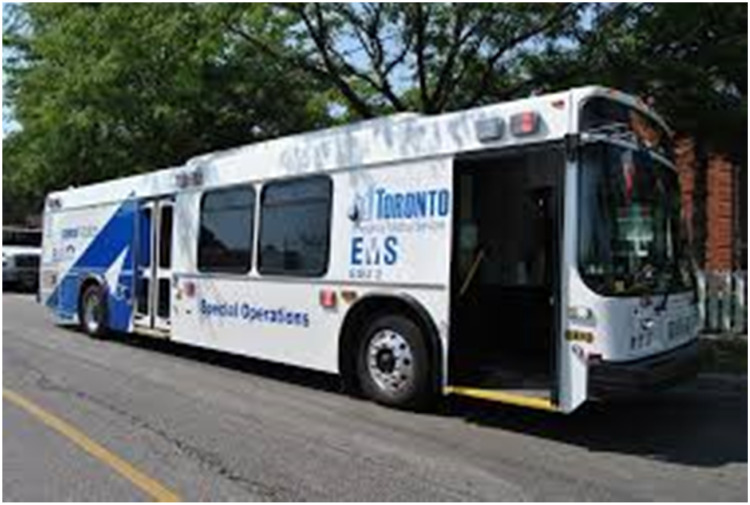
Southern surge planning with Toronto Paramedic Services.

In Southern Ontario, Ornge has partnered with Toronto Paramedic Services (TPS) to develop a plan for transporting multiple critically ill COVID-19 patients in the TPS ambulance bus (Figure 2). This ambulance bus would be driven by a TPS paramedic, and critical care could be provided by OSRT paramedics. The bus can transport 4 ventilated patients or up to 8 stretcher-bound patients.

## UNCONVENTIONAL TASKS DURING THE COVID-19 PANDEMIC

Unforeseen challenges have arisen as a result of the pandemic that threaten traditional health care processes. COVID-19 has temporarily collapsed the commercial air industry.^14^ Unfortunately, rural and remote communities depend on these flights for many health care processes including laboratory testing. As of April 30, Ornge has transported 450 COVID-19 test samples and five bone marrow biopsy samples. Going forward, Ornge is exploring the use of drone technology with stakeholders as a potential solution to this gap.

Ornge is also contributing in other ways. Ornge is providing administrative and technical support to the provincial critical care command center, tasked to manage critical care capacity. Additionally, Ornge, in conjunction with partners, has offered its 24/7 Transport Medicine Physicians (TMPs) to provide virtual consultations to any hospital provider in the province.

## CONCLUSION

Air ambulances and critical care transport providers are operationally-focused organizations that can play a critical role during pandemics. We have provided a short synopsis of our experience during the COVID-19 pandemic so that other organizations can learn from our experiences.
